# Ventriculoperitoneal shunt insertion for hydrocephalus in human immunodeficiency virus-infected adults: a systematic review and meta-analysis protocol

**DOI:** 10.1186/s13643-017-0603-7

**Published:** 2017-10-16

**Authors:** James J. M. Loan, Ncedile Mankahla, Graeme Meintjes, A. Graham Fieggen

**Affiliations:** 1 College of Medicine and Veterinary Medicine, University of Edinburgh, Edinburgh, UK; 2 Institute of Neurosciences, 1345 Govan Road, Glasgow, G51 4TF UK; 3 Groote Schuur Hospital, Observatory, Cape Town, South Africa; 4 University of Cape Town, Rondebosch, Cape Town, South Africa; 5 Red Cross War Memorial Children’s Hospital, Rondebosch, Cape Town, South Africa

## Abstract

**Background:**

Hydrocephalus is a recognised complication of human immunodeficiency virus (HIV)-related opportunistic infections. Symptomatic raised cerebrospinal fluid pressure can be treated with ventriculoperitoneal shunt insertion (VPS). In HIV-infected patients however, there is a concern that VPS might be associated with unacceptably high rates of mortality. We aim to systematically review and appraise published literature to determine reported outcomes and identify predictors of outcome following VPS in relevant subgroups of HIV-infected adults.

**Methods:**

The following electronic databases will be searched: The Cochrane Central Register of Controlled Trials (CENTRAL), MEDLINE (PubMed), EMBASE, CINAHL (EBSCOhost), LILACS (BIREME), Research Registry (www.researchregistry.com), the metaRegister of Controlled Trials (mRCT) (www.controlled-trials.com), ClinicalTrials.gov (www.clinicaltrials.gov) and OpenSIGLE database. Any randomised studies, cohort studies, case-control studies, interrupted time series or sequential case series reporting survival following VPS in HIV-infected individuals will be included. If high-quality homogenous studies exist, meta-analysis will be conducted to determine 1-, 6- and 12-month mortality with comparison made between underlying aetiologies of hydrocephalus.

**Discussion and conclusion:**

This study will generate a comprehensive review of VPS in HIV-infected patients for publication. The primary outcome of meta-analysis is 12-month survival. If only low-quality, heterogeneous studies are available, this study will demonstrate this deficiency and will be of value in justifying and aiding the design of future studies.

**Systematic review registration:**

PROSPERO CRD42016052239

**Electronic supplementary material:**

The online version of this article (10.1186/s13643-017-0603-7) contains supplementary material, which is available to authorized users.

## Background

### Description of the condition

Human immunodeficiency virus (HIV) is a lentivirus and is the causative organism for the acquired immunodeficiency syndrome (AIDS) [1]. Two major subtypes of HIV—HIV-1 and HIV-2—are known to exist [2]. Over 95% of patients infected with HIV will, without treatment, develop progressive immunodeficiency as indicated by falling CD4-positive T cell counts and/or development of AIDS defining illnesses within 10 years of seroconversion [3, 4]. In general, disease progression is slower in those infected with HIV-2 compared with the globally more common HIV-1 [1, 2].

HIV infection is associated with myriad central nervous system (CNS) sequelae. These include, but are not limited to, toxoplasmosis, primary CNS lymphoma (PCNSL), tuberculous meningitis (TBM), cryptococcal meningitis and immune reconstitution inflammatory syndrome (IRIS) [5–8]. These may all disrupt cerebrospinal fluid (CSF) circulation causing an abnormal accumulation of CSF within the cranium, an associated rise in intracranial pressure and consequent cerebral injury. This condition is called hydrocephalus. In communicating hydrocephalus, ventricular CSF is continuous with CSF in the subarachnoid space. Communicating hydrocephalus develops because of excessive CSF production or reduced CSF resorption [9]. In non-communicating hydrocephalus, CSF passage from the ventricles to subarachnoid space is occluded. With time and successful treatment of the primary pathology, acute hydrocephalus might resolve, with normalisation of CSF pressure, or become associated with chronically raised CSF pressure [10]. Age-related cerebral tissue loss as well as HIV-associated neurocognitive disorder are associated with enlargement of CSF spaces due to generalised brain atrophy and may result in the radiological appearance of hydrocephalus but are associated with normal CSF pressure and flow, so-called hydrocephalus ex vacuo [9, 11]. This is not an indication for surgical treatment [9].

Meningeal infections including TBM, cryptococcal meningitis and bacterial meningitis can impair CSF resorption at arachnoid granulations, resulting in communicating hydrocephalus [10, 12]. TBM, which is associated with thick basal exudate, can also result in non-communicating hydrocephalus [13]. TBM in the context of HIV infection appears to be associated with substantially increased morbidity and mortality compared with HIV non-infected patients [14, 15]. This might arise because of synergistic effects of HIV and tuberculosis in the CNS causing depressed monocyte and microglial mediated immunity and dysregulated cytokine production [16–21]. Hydrocephalus in TBM frequently persists following successful antimicrobial treatment [22]. Conversely, the majority of HIV-infected patients with raised CSF pressures secondary to cryptococcal meningitis (typically communicating hydrocephalus) experience normalising of CSF pressure and resolution of hydrocephalus following successful antimicrobial therapy [10]. A small proportion does require temporary CSF diversion for initial acute hydrocephalus [23]. Mass lesions such as toxoplasmosis, PCNSL, metastases from HIV-associated non-CNS malignancy and tuberculoma can trigger the development of non-communicating hydrocephalus by occlusion of ventricular outflow pathways [24, 25]. In these instances, hydrocephalus resolution post-treatment is dependent on restoration of normal ventricular outflow pathway anatomy. IRIS affecting the CNS is defined by a transient worsening or development of new inflammatory lesions during immune reconstitution around infective foci and might also present with hydrocephalus secondary to meningitis or mass lesions [26, 27].

### Epidemiology

As of 2015, it was estimated that 36.7 million people globally were living with HIV infection [28]. Of these, 19.0 million live in Eastern and Southern Africa [28]. HIV infection disproportionately affects certain vulnerable populations including intravenous substance misusers, sex workers and their clients, men who have sex with men and transgender people [28]. The risk of *Mycobacterium tuberculosis* disease is estimated to be 20 times greater in HIV-infected patients compared with their HIV non-infected counterparts, an effect which is most pronounced in low-income populations [29, 30]. As such, one cadaveric study conducted in the Ivory Coast in 1991 found that 32% of in-hospital deaths of HIV-infected patients were a result of tuberculosis with TBM affecting 20% of these [31]. The increasing availability of antiretroviral therapy (ART) has seen a decline in the incidence of new opportunistic intracranial infections, PCNSL and reduced AIDS-related deaths [5, 32, 33]. However, with improved survival and ongoing HIV transmission, the global prevalence of HIV and the associated burden of chronic disease continues to rise [28].

The incidence of hydrocephalus associated with HIV infection is poorly quantified at present. Small studies comparing clinical and radiological characteristics of HIV-associated and HIV non-associated patients with conditions associated with hydrocephalus provide some information: HIV-associated TBM appears to be associated with lower rates of non-communicating hydrocephalus than HIV non-associated TBM [34], and in some studies, over 50% of patients with cryptococcal meningitis, which occurs almost exclusively in HIV-infected patients, have exhibited raised CSF pressures [10]. However, non-communicating hydrocephalus is uncommon in cryptococcal meningitis [10, 35]. It is also important to note that any other cause of hydrocephalus may also be seen in the HIV-infected population; hence, adults might suffer the ongoing effects of infant-onset or congenital hydrocephalus. This is estimated to affect 1.1 in 1000 infants and 32 per 10,000 births and may be due to anatomical abnormalities such as neural tube defects and Chiari malformations or arise secondary to perinatal insults such as intraventricular haemorrhage and bacterial meningitis [36]. In HIV non-infected high-income populations, intracranial haemorrhage and malignancy account for 45 and 30% of new cases of adult onset hydrocephalus, respectively [37]. A lack of dedicated epidemiological research concerning HIV-associated hydrocephalus makes data from these small studies difficult to generalise, and little is known about the distribution of non-infective causes of hydrocephalus associated with HIV.

### Description of the intervention

A ventriculoperitoneal shunt (VPS) is a surgically implanted device that allows flow of fluid from the cerebral ventricles into the peritoneal cavity. It consists of a silicone ventricular catheter that is inserted through a single burr hole, using either image guidance or anatomical landmarks to locate the ventricle. The catheter is sometimes rifampicin and clindamycin or silver impregnated [38]. It is connected to a valve that is usually placed in the subgaleal space. The valve can be pressure or flow regulated and may contain an antisiphon device [39]. This is connected to a length of distal tubing that is tunnelled subcutaneously from the valve site to the level of the abdomen where it is inserted through the abdominal wall into the peritoneal cavity where the distal catheter tip sits.

A functioning VPS provides a permanent pathway for ventricular CSF outflow and allows for normalisation of CSF pressure in hydrocephalus. Common complications following successful VPS include shunt infection, ventriculitis, proximal catheter occlusion or migration, shunt fracture, over drainage and distal catheter occlusion [40]. In one US cohort, 21% of adults who underwent VPS between 1990 and 2000 suffered a complication within the first year and 26% required replacement or removal of the shunt during the study period [41]. One cohort of mostly paediatric patients who underwent VPS between 2004 and 2007 in Kenya, a low-middle-income country with high HIV prevalence, there was a 2-year cumulative malfunction rate of 35% with 59.8% having “poor outcome” and 8.5% mortality [42–44]. However, follow-up was reported as incomplete and this might not therefore been an underestimate of the complication rate in such a setting.

Alternatives to VPS include ventriculoatrial shunting where the distal catheter tip is sited in the right cardiac atrium and ventriculopleural shunting where it is sited in the pleural cavity. In some instances, an external ventricular drain (EVD) may be sited in preference to a shunt [39]. For this, a ventricular catheter is placed and the distal tip externalised through the scalp and connected without a valve to a drain mounted at a specific height above the tragus of the ear. This allows gravity to limit CSF flow via the catheter below a desired pressure. However, this is only a temporary means of CSF diversion. For some cases of communicating hydrocephalus, it may be sufficient to drain CSF through serial lumbar puncture or a lumbar drain [39]. Lumbar drains may be externalised using a similar system to an EVD or tunnelled and have the distal tubing placed in the peritoneal cavity—a lumboperitoneal shunt [39]. In cases of non-communicating hydrocephalus, it is sometimes possible to surgically divide the lamina terminalis or endoscopically conduct a third ventriculostomy (ETV) to allow for flow of ventricular CSF to the subarachnoid space [13]. However, this procedure is challenging—particularly in TBM [13]—and requires significant expertise and equipment that may not be available in emergency or resource-limited settings.

### Why it is important to do this review

A proportion of patients infected with HIV develop hydrocephalus as a complication of CNS opportunistic infections. As described above, infectious aetiologies of hydrocephalus are over-represented in HIV-infected patients and HIV directly impacts on the pathological processes in intracranial infection. The literature from HIV non-infected patient populations therefore cannot be generalised to those with HIV infection.

VPS can be immediately life-saving in acute hydrocephalus and may be the sole treatment option for patients who are dependent on CSF diversion following emergency EVD insertion and are not candidates for ETV. However, two of the most cited studies comparing VPS in HIV-infected patients with TBM to HIV non-infected counterparts demonstrated mortality rates in excess of 60% in HIV-infected patients [45, 46]. Small numbers of HIV-infected patients in these studies achieved good outcome following VPS [45, 46]. Better outcomes have been reported following VPS insertion for other causes of HIV-associated hydrocephalus [47, 48]. To determine if the existing literature is sufficient to inform selection of HIV-infected patients for VPS in clinical practice, it is necessary for a systematic review to be conducted.

### Objectives

Our objectives were to compare post-VPS mortality, VPS infection, VPS malfunction and clinical outcome and rates of other complications at 1, 6 and 12 months between different aetiologies of HIV-associated hydrocephalus using quantitative meta-analysis and production of a narrative review. They would allow the identification of baseline patient characteristics which are predictive of good outcome following VPS in HIV infection.

## Methods

This protocol was produced with reference to the PRISMA-P checklist (see Additional file 1).

### Inclusion criteria

#### Types of studies

We will include the following study designs: randomised clinical trials (RCTs), one- and two-group cohort studies, case-control studies, interrupted time series and consecutive case series. Descriptive, non-comparative studies of relevant populations will be eligible for inclusion. Studies reporting data on a population with a subset of HIV-infected individuals will only be included if outcome data on the subpopulation of HIV-infected patients is reported.

#### Types of participants

All studies of patients aged 16 years or older who have undergone a VPS procedure and have a positive serological diagnosis of HIV infection or a detectible HIV viral load will be included, regardless of the presence of any other complicating CNS pathology.

#### Types of interventions

Studies of patients who have undergone a VPS procedure using any type of catheter, valve or insertion technique will be included.

#### Types of outcome measures

We will include studies of primary data that report measures of overall survival, AIDS-specific mortality or VPS failure. We define VPS failure in all patient groups as any complication requiring shunt removal or revision. Studies reporting perioperative complications of VPS will additionally be included for secondary analysis.

### Outcomes

Our study’s primary outcome will be 1-year post-VPS survival with comparison between all identified aetiologies of hydrocephalus in HIV-infected patients treated with VPS. We define separate aetiologies as being different sites of anatomical abnormality, with congenital and acquired causes considered separate: subarachnoid haemorrhage, intraventricular haemorrhage and different suspected or proven pathogens for infectious causes. Secondary outcomes will be survival at 1 and 6 months andAIDS-specific mortality, VPS failure, rates of perioperative complications and measures of outcome using validated outcome measures [49–51] at 1 month, 6 months and 1 year post-VPS insertion. For all measures, comparison will be made between aetiologies of hydrocephalus.

### Search methods for the identification of studies

#### Scoping searches

Scoping searches have been undertaken to identify candidate studies and inform keyword selection for electronic searching [15, 45–47].

#### Electronic searches

We will search the following electronic databases: The Cochrane Central Register of Controlled Trials (CENTRAL), MEDLINE (PubMed), EMBASE, CINAHL Plus (EBSCOhost), LILACS (BIREME), Research Registry (www.researchregistry.com), the metaRegister of Controlled Trials (mRCT) (www.controlled-trials.com), ClinicalTrials.gov (www.clinicaltrials.gov) and African Journals Online (AJOL). Grey literature searching will be performed using the OpenGREY database. There will be no date restrictions in electronic searches. Our search sensitivity has been ensured by confirming that candidate studies identified during scoping searches are included in the search yields. See Appendix 1 for full search strategies.

#### Searching other resources

We will examine the reference lists of included studies to identify any additional studies for inclusion. Experts in relevant fields will be consulted and asked to identify key studies. Any studies identified for consideration of inclusion by these consults that were not detected by our search will be documented in our final report. We will not hand search any conference proceedings or journals. Disagreement will be resolved through consensus discussion and/or consideration of a third author (GF).

### Data extraction and management

Data will be extracted using standardised proformas (Appendix 2) and entered into review manager [52] by two authors (JL, NM).We will use the software package EndNote for reference management [53].

### Data collection and analysis

#### Selection of studies

Two review authors (JL, NM) will independently screen all titles and abstracts yielded by the literature search. During screening, studies will be scrutinised against inclusion criteria. Those not meeting criteria will be excluded. For those which meet the criteria or for which it is impossible to tell if criteria are met on the basis of title and abstract the full manuscript will be sought and reviewed. Any subsequent exclusions will be documented.

#### Assessment of risk of bias in included studies

Each included study will be independently assessed for risk of bias by two study authors (JL, NM). Randomised studies will be assessed using the ‘Risk of bias’ tool from Chapter 8.5 of the *Cochrane Handbook of Systematic Reviews of Intervention* [54]. Non-randomised studies (NRS) will be assessed using the Canadian National Collaborating Centre for Methods and Tools “Quality Assessment Tool for Quantitative Studies” [55, 56]. Summary outcomes of these assessments will be presented.

### Measures of effect

In the narrative review, we will summarise outcomes as reported by each included study separately. If data comparing outcome across different aetiologies of hydrocephalus exists, we will report it. If meta-analysis is performed, pooled odds ratios (OR) will be reported. The primary outcome of any RCTs comparing VPS with any other management strategy will be reported. If multiple trials of the same interventions report similar outcomes, meta-analysis will be conducted according to guidance by the *Cochrane Handbook of Systematic Reviews of Interventions* [54].

### Dealing with missing data

We will attempt to contact the authors of included studies electronically for any unreported details. If, following this, uncertainty remains, we will report available data and state missing data. Studies with missing mortality data will not be eligible for inclusion in meta-analysis.

### Assessment of heterogeneity

On the basis of extracted data, studies will be compared for clinical and methodological heterogeneity. Where apparently homogenous studies report similar outcome measures at similar time points, the *I* ^2^ (%) and *T* ^2^ statistics will be calculated, with *I* ^2^ greater than 50% suggesting substantial heterogeneity. For estimation of statistical heterogeneity, we will use odds of mortality between aetiologies of HIV-associated hydrocephalus as the measure of effect.

### Data synthesis

If substantial clinical, methodological or statistical heterogeneity is detected or if no comparative studies are included, we will not attempt meta-analysis and will present solely a narrative review. If homogenous comparative studies are identified, then meta-analysis of pooled mortality data will be conducted using the Peto method. OR at 12, 6 and 1 month post-VPS between pooled populations with different aetiologies of hydrocephalus will be compared. If greater than 10 homogenous studies are included for meta-analysis, meta-regression will be attempted. Covariables for this will include, where reported, age categories (16–24; 25–44; 45–59; ≥ 60), CD4^+^ cell count, time from presentation to VPS, initial Glasgow Coma Score [50], and shunt catheter type—either conventional, antibiotic or silver impregnated. Where not all variables are reported, regression will be conducted using the maximum covariables reported by all eligible studies. Where meta-analysis is performed, sensitivity analysis will be undertaken to determine the relative effects of inclusion of each individual study, as well as RCTs and non-randomised studies from different World Bank income categories: low-income, lower-middle-income, upper-middle-income and high-income economies. GRADEpro will be used to summarise strength of available data [57].

### Protocol amendments

This is the original study protocol. Any subsequent protocol amendments will be published on the PROSPERO database of systematic reviews. Amendments to the protocol manuscript following peer review are documented (see Additional file 2).

## Discussion

HIV infection and HIV-associated hydrocephalus disproportionately impact on patients from low-middle-income countries [28]. Unfortunately, it is believed that the neurosurgical management of CNS complications of HIV infection has received relatively little research attention, and consequently, guidelines for management to date have been based on low-quality evidence [58, 59]. As outcome following VPS in HIV appears to be somewhat dependent on population income, it is important that studies of outcome take into account population demographics and that the study of low-middle-income populations is urgently prioritised [41, 42]. This study will begin to address this need by summarising existing evidence to inform clinical practice in relevant populations where the data supports this. We also will define deficiencies in existing literature and to guide research direction towards meaningfully answering relevant questions.

### Additional files

Additional file 1: PRISMA-P Checklist. Standardised reporting guideline for production of systematic review protocol. (PDF 184 kb)

Additional file 2: Responses to peer reviewer comments. HIV VPS Protocol Reviewers Responses. (DOCX 126 kb)

## Appendix 1

### Search terms

#### MEDLINE (PubMed)

Abbreviated search strategy (full syntax available)

1. human
2. immunodeficiency
3. virus
4. 1 AND 2 AND 3
5. human immunodeficiency virus
6. HIV
7. acquired
8. immunodeficiency
9. 7 AND 8
10. acquired immunodeficiency syndrome
11. AIDS
12. 4 OR 5 OR 6 OR 9 OR 10 OR 11
13. ventriculoperitoneal
14. ventric*
15. ventricular
16. 14 OR 15
17. peritone*
18. peritoneal
19. 17 OR 18
20. 16 AND 19
21. VP
22. cerebrospinal
23. cerebrosp*
24. CSF
25. 22 OR 23 OR 24
26. 13 OR 20 OR 21 OR 25
27. shunt*
28. shunt
29. catheter*
30. catheter
31. diversion
32. divers*
33. 27 OR 28 OR 29 OR 30 OR 31 OR 32
34. 26 AND 33
35. ventriculoperitoneal shunt
36. 34 OR 35
37. 12 AND 36

#### CENTRAL

1. Human
2. Immunodeficiency
3. Virus
4. 1 and 2 and 3
5. MeSH descriptor: [HIV] explode all trees
6. Human immunodeficiency virus
7. HIV
8. acquired
9. Immunodeficiency
10. 8 and 9
11. acquired immunodeficiency syndrome
12. AIDS
13. MeSH descriptor: [Acquired Immunodeficiency Syndrome] explode all trees
14. #4 or #5 or #6 or #7 or #10 or #11 or #12 or #13
15. ventriculoperitoneal
16. ventricular
17. peritoneal
18. 16 and 17
19. VP
20. cerebrospinal
21. CSF
22. MeSH descriptor: [Cerebrospinal Fluid] explode all trees
23. 15 or 18 or 19 or 20 or 21 or 22
24. shunt
25. MeSH descriptor: [Cerebrospinal Fluid Shunts] explode all trees
26. catheter
27. MeSH descriptor: [Catheters] explode all trees
28. diversion
29. #24 or #25 or #26 or #27 or #28
30. 23 and 29
31. ventriculoperitoneal shunt
32. MeSH descriptor: [Ventriculoperitoneal Shunt] explode all trees
33. 30 or 31 or 32
34. 14 and 33

#### EMBASE

Unlimited search terms linked to Subject Heading

1. human/
2. immunodeficiency.mp. or immune deficiency/
3. virus/
4. 1 and 2 and 3
5. human immunodeficiency virus.mp. or Human immunodeficiency virus/
6. HIV.mp. or Human immunodeficiency virus/
7. acquired.mp.
8. immunodeficiency.mp. or immune deficiency/
9. 7 and 8
10. acquired immunodeficiency syndrome.mp. or acquired immune deficiency syndrome/
11. AIDS.mp. or acquired immune deficiency syndrome/
12. 4 or 5 or 6 or 9 or 10 or 11
13. cerebrospinal fluid shunting/ or shunting/ or shunt infection/ or hydrocephalus/ or brain ventricle peritoneum shunt/ or ventriculoperitoneal.mp. or cerebrospinal fluid/
14. ventricular.mp
15. brain ventricle peritoneum shunt/ or hydrocephalus/ or ventriculoatrial shunt/ or ventric*.mp.
16. 14 or 15
17. peritoneum/ or peritone*.mp.
18. peritoneal catheter/ or peritoneal drain/ or peritoneal.mp. or peritoneal cavity/
19. 17 or 18
20. 16 and 19
21. vp.mp.
22. cerebrospinal fluid level/ or cerebrospinal.mp. or cerebrospinal fluid drainage system/ or cerebrospinal fluid analysis/ or cerebrospinal fluid/ or cerebrospinal fluid shunting/ or cerebrospinal fluid drainage/
23. cerebrospinal fluid/ or cerebrosp*.mp
24. CSF.mp. or cerebrospinal fluid/
25. 22 or 23 or 24
26. 13 or 20 or 21 or 25
27. brain ventricle peritoneum shunt/ or shunt.mp. or shunt occlusion/ or ventriculoatrial shunt/ or shunt thrombosis/ or shunt failure/ or shunt infection/
28. shunt*.mp.
29. catheter.mp. or catheter fracture/ or intrathecal catheter/ or drainage catheter/ or catheter infection/ or neurological catheter/ or catheter care/ or catheter leakage/ or subdural catheter/ or peritoneal catheter/ or catheter occlusion/ or catheter sheath/ or catheter thrombosis/ or ventriculostomy catheter/ or catheter/ or catheter dislocation/ or antimicrobial catheter/ or catheter migration/ or catheter complication/ or catheter valve/ or intracranial catheter/
30. catheter/
31. diversion.mp.
32. divers*.mp.
33. 27 or 28 or 29 or 30 or 31 or 32
34. 26 and 33
35. ventriculoperitoneal shunt.mp. or brain ventricle peritoneum shunt/
36. 34 or 35
37. 12 and 36

#### CINAHL Plus (EBSCOhost)

S1. MW Human immunodeficiency virus.

S2. TI Human AND TI Immunodeficiency AND TI virus.

S3. (MH “HIV-Infected Patients”) OR (MH “HIV Protease Inhibitors”) OR (MH “HIV Seropositivity”) OR (MH “AIDS Serodiagnosis”).

S4. TX acquired OR TX immunodeficiency.

S5. MW aquired immunodeficiency syndrome OR TX AIDS.

S6. S1 OR S2 OR S3 OR S4 OR S5.

S7. TX ventric* OR TX ventricular.

S8. TX peritoneal AND TX peritone*.

S9. S7 AND S8.

S10. TX ventriculoperitoneal.

S11. TX VP.

S12. TX cerebrospinal OR MW cerebrospinal OR TX cerebrosp*.

S13. TX CSF.

S14. S12 OR S13.

S15. S9 OR S10 OR S11 OR S14.

S16. TX shunt* OR MW shunt.

S17. TX catheter* OR MW catheter.

S18. MW diversion OR TX divers*.

S19. S16 OR S17 OR S18.

S20. S15 AND S19.

S21. TX ventriculoperitoneal shunt OR MW ventriculoperitoneal shunt.

S22. S20 OR S21.

S23. S6 AND S22

#### LILACS (VHL)

((((human) AND (immunodeficiency) AND (virus)) OR (human immunodeficiency virus) OR (HIV) OR ((acquired) AND (immunodeficiency)) OR (acquired immunodeficiency syndrome) OR (AIDS))) AND (((ventriculoperitoneal) OR (((ventric*) OR (ventricular)) AND ((peritone*) OR (peritoneal))) OR (VP) OR ((cerebrospinal) OR (cerebrosp*) OR (CSF)))) AND ((shunt*) OR (shunt) OR (catheter*) OR (catheter) OR (diversion) OR (divers*) OR (ventriculoperitoneal shunt))

#### Research Registry (www.researchregistry.com)

Individual searches for:

1. HIV
2. AIDS
3. acquired immunodeficiency syndrome
4. Human immunodeficiency
5. ventriculoperitoneal
6. hydrocephalus
7. cerebrospinal
8. CSF
9. shunt

#### The metaRegister of Controlled Trials (mRCT) (www.controlled-trials.com)

((((human) AND (immunodeficiency) AND (virus)) OR (human immunodeficiency virus) OR (HIV) OR ((acquired) AND (immunodeficiency)) OR (acquired immunodeficiency syndrome) OR (AIDS))) AND (((ventriculoperitoneal) OR (((ventric*) OR (ventricular)) AND ((peritone*) OR (peritoneal))) OR (VP) OR ((cerebrospinal) OR (cerebrosp*) OR (CSF)))) AND ((shunt*) OR (shunt) OR (catheter*) OR (catheter) OR (diversion) OR (divers*) OR (ventriculoperitoneal shunt))

#### ClinicalTrials.gov (www.clinicaltrials.gov)

(HIV OR (Human immunodeficiency virus) OR AIDS) AND ((Ventriculoperitoneal Shunt) OR (CSF diversion) OR Hydrocephalus)

#### OpenGREY

((((human) AND (immunodeficiency) AND (virus)) OR (human immunodeficiency virus) OR (HIV) OR ((acquired) AND (immunodeficiency)) OR (acquired immunodeficiency syndrome) OR (AIDS))) AND (((ventriculoperitoneal) OR (((ventric*) OR (ventricular)) AND ((peritone*) OR (peritoneal))) OR (VP) OR ((cerebrospinal) OR (cerebrosp*) OR (CSF)))) AND ((shunt*) OR (shunt) OR (catheter*) OR (catheter) OR (diversion) OR (divers*) OR (ventriculoperitoneal shunt))

#### African Journals Online (AJOL)

(((human immunodeficiency virus) OR (acquired immunodeficiency syndrome) OR (AIDS))) AND (((ventriculoperitoneal) OR (((ventric*) OR (VP) OR ((cerebrosp*) OR (CSF)))) AND ((shunt*) OR (catheter*) OR (divers*) OR (ventriculoperitoneal shunt))

## Appendix 2

**Table 1:** Data extraction proforma

	Data to be extracted	Data
Population	Age range	
	Diagnostic modality for hydrocephalus/CSF hypertension	
	Diagnostic modality for underlying aetiology	
	CD4^+^ cell count range (for each underlying aetiology)	
	Country population derived from	
	Severity of disease at baseline (using GCS, palur grade, MRC scale, GOS or mRS) for each underlying aetiology	
	Communicating or non-communicating hydrocephalus	
Intervention	Primary VPS or following EVD	
	Catheter type (plain silicon, antibiotic impregnated, silver impregnated)	
Comparison intervention	Description of intervention	
	Frequency of intervention	
Outcome (for each underlying aetiology and intervention)	Survival at 1, 6 and 12 months	
	Causes of death at 1, 6 and 12 months	
	Rate of shunt failure at 1, 6 and 12 months	
	Rates of complication at 1, 6 and 12 months	
	Validated outcome measure at 1, 6 and 12 months	

## Abbreviations

AIDS: Acquired immunodeficiency syndrome
ART: Antiretroviral therapy
CD4: Cluster of differentiation 4
CNS: Central nervous system
CSF: Cerebrospinal fluid
ETV: Endoscopic third ventriculostomy
EVD: External ventricular drain
HIV: Human immunodeficiency virus
IRIS: Immune reconstitution inflammatory syndrome
PCNSL: Primary central nervous system lymphoma
RCT: Randomised clinical trial
TBM: Tuberculous meningitis
VPS: Ventriculoperitoneal shunt
