# Supplementary material for: Ventriculoperitoneal shunt insertion for hydrocephalus in human immunodeficiency virus-infected adults: a systematic review and meta-analysis protocol
Source: Syst Rev. 2017 Oct 16;6:201. doi: 10.1186/s13643-017-0603-7 (PMC5644263; doi:10.1186/s13643-017-0603-7)
Supplement: Supplementary file 2 — Responses to peer reviewer comments. HIV VPS Protocol Reviewers Responses. (DOCX 126 kb) [file 13643_2017_603_MOESM2_ESM.docx]

# Ventriculoperitoneal shunt insertion in human immunodeficiency virus infected adults: A systematic review and meta-analysis.

## Responses to reviewer comments

### Authors

James JM. Loan,^1,2,3^ Ncedile Mankahla,^3,4,5^ Graeme Meintjes,^3,4^ A. Graham Fieggen^1,3,4,5^

### Affiliations

1) College of Medicine and Veterinary Medicine, University of Edinburgh, Edinburgh, UK
2) Aberdeen Royal Infirmary, Foresterhill Road, Aberdeen, UK
3) Groote Schuur Hospital, Observatory, Cape Town, South Africa
4) University of Cape Town, Rondebosch, Cape Town, South Africa
5) Red Cross War Memorial Children’s Hospital, Rondebosch, Cape Town, South Africa

| Reviewer comment | Response |
| --- | --- |
| L1, of the salient concepts, "hydrocephalus" is not highlighted in the title! | Title adjusted |
| L21, What CSF stands for?? It is the first occurrence! | CSF stands for Cerebrospinal fluid. This has been ammended |
| L42, Discussion and conclusion | Heading updated |
| L60 Epidemiology of hydrocephalus will value this section. At least epidemiology of hydrocephalus on HIV-infected adults! | We have added a new description of what data is available: “The incidence of hydrocephalus associated with HIV infection is poorly quantified at present. Small studies comparing clinical and radiological characteristics of HIV associated and HIV non-associated patients with conditions associated with hydrocephalus provide some information: HIV-associated TBM appears to be associated with lower rates of non-communicating hydrocephalus than HIV non-associated TBM(34), and in some studies, over 50% of patients with cryptococcal meningitis, which occurs almost exclusively in HIV-infected patients, have exhibited raised CSF pressures(10). However, non-communicating hydrocephalus is uncommon in cryptococcal meningitis(10, 35). It is also important to note that any other cause of hydrocephalus may also be seen in the HIV-infected population, hence adults might suffer the ongoing effects of infant-onset or congenital hydrocephalus. This is estimated to affect 1.1 in 1000 infants and 32 per 10,000 births and may be due to anatomical abnormalities such as neural tube defects and Chiari malformations or arise secondary to perinatal insults such as intraventricular haemorrhage and bacterial meningitis(36). In HIV non-infected high-income populations intracranial haemorrhage and malignancy account for 45% and 30% of new cases of adult onset hydrocephalus, respectively(37). A lack of dedicated epidemiological research concerning HIV-associated hydrocephalus makes data from these small studies difficult to generalize, and little is known about the distribution of non-infective causes of hydrocephalus associated with HIV.” |
| L128 Objectives  Invert to 1, 6, and 12 months.  Base on the taxonomy in building your objectives, the second and the third are clearly embed in the first. Need reframing, otherwise. | Updated order of months. Objectives consolidated. |
| L143, Why setting 16 y.o as cut-off? | The aetiologies, disease processes, and consequently anticipated clinical outcomes in paediatric HIV-associated hydrocephalus differ vastly from the adult population. Further, means of outcome assessment in the very young are different. Comparison of data from paediatric populations with adults would therefore be misleading. A review of paediatric studies should be undertaken as a separate review with appropriate outcomes for use in paediatric populations. |
| L143, Discretionary comment: It would be more valuable to consider all age participants. Comparison could be made then. | See above. |
| L145, in reference to the objective or the sentence before, "…had any or no other intracranial…" this is unclear!! | This has been clarified |
| L150, the types of outcome is well set, however, author shall inform on why only "studies where the shunt has been inserted using image guidance or anatomical landmark techniques" only, are  included. | Image guidance and anatomical landmark techniques are the only means of insertion that the authors are aware of. The outcome has been clarified to reflect this. |
| L153, does "VPS survival" means " or VPS survival on HIV infected adults"??? | Updated to VPS failure: We will include studies of primary data that report measures of overall survival, AIDS specific mortality or VPS failure. We define VPS failure in all patient groups as any complication requiring shunt removal |
| L161, "…and one year post-VPS insertion…" is already mentioned at L 157; otherwise, clarification is necessary! | This has been clarified: “Secondary outcomes will be survival at one and six months as well as, AIDS specific mortality, VPS failure, rates of perioperative complications and measures of outcome using validated outcome measures(45-47) at one month, six months and one year post-VPS insertion.” |
| L185, sentence to be reviewed. | Sentence adjusted: “We will use the software package EndNote for reference management.(49)” |
| L 226, Please, add targeted categories! | These have been added: “…RCTs and non-randomised studies from different World Bank income categories; low income, lower-middle income, upper-middle income, and high income economies.” |
| Objectives  What do different aetiologies of HIV-associated hydrocephalus include? | We have updated our outcomes section to fully define different aetiologies of HIV associated hydrocephalus: “We define separate aetiologies as being different sites of anatomical abnormality, with congenital and acquired causes considered separate; subarachnoid haemorrhage; intraventricular haemorrhage; different suspected or proven pathogens for infectious causes.” |
| Types of studies  Should eligible studies compare mortality following ventriculoperitoneal shunt (VPS) between different aetiologies of HIV-associated hydrocephalus? | As it is anticipated that few of these studies will exist, we will include non-comparative studies of a single aetiology. These studies will feed into understanding survival in these subgroups, however, they will not be eligible for inclusion in comparative meta-analysis. “Descriptive, non-comparative studies of relevant populations will be eligible for inclusion.”  We have amended our data synthesis section to clarify this. |
| Assessment of risk of bias in included studies  Authors may consider using the ROBINS-I tool (Risk Of Bias In Non-randomized Studies - of Interventions) for evaluating the risk of bias in the results of non-randomized studies. The tool is described in (1).  1. Sterne JA, Hernán MA, Reeves BC, Savović J, Berkman ND, Viswanathan M, et al. ROBINS-I: a tool for assessing risk of bias in non-randomised studies of interventions. BMJ. 2016 Oct 12;355:i4919. | The ROBINS-I tool described is currently unvalidated, unlike the Quality Assessment Tool for Quantitative Studies, which is an established tool and was favourably assessed by a health technology assessment review (2). Further, it is designed for the assement of interventions and lacks flexibility in this regards. By comparison the Quality assement tool for quantitative studies is designed for assessments of population level studies and has greater flexibility. We therefore think that our approach is currently the optimum, although we acknowledge that this might change in the future should the ROBINS-I tool be sucessfully validated.  2. Deeks JJ, Dinnes J, D'Amico R, Sowden AJ, Sakarovitch C, Song F, Petticrew M, Altman DG; International Stroke Trial Collaborative Group; European Carotid Surgery Trial Collaborative Group. Evaluating non-randomised intervention studies. Health Technol Assess. 2003;7(27):iii-x, 1-173. |
| Assessment of heterogeneity  It would be useful to report the estimated tau apart from the I^2 statistic. In that case, please report the estimator that will be used for heterogeneity. | Where random effects Mantel-Haenszel testing is used, we will report the T2 statistic additionally. We have updated our methods of assessment of heterogeneity and meta-analysis to reflect this.  Additionally, we have added: “For estimation of statistical heterogeneity, we will use odds of mortality between aetiologies of HIV-associated hydrocephalus as the measure of effect” |
| Data synthesis  1. Authors say that they will perform meta-regression if "greater than 10 homogeneous studies are included for meta-analysis". What will be the characteristics to be investigated? The different aetiologies are the comparators in this setting; how can they be also covariates in the meta-regression model?  2. I believe that the description of the sensitivity analyses to be performed need to be more specific. | 1. We have re-written the methodology on meta-regression: “If greater than 10 homogenous studies are included for meta-analysis, meta-regression will be attempted. Covariates for this will include, where reported, age categories (16-24; 25-44; 45-59; ≥60); CD4+ cell count; time from presentation to VPS; initial Glasgow Coma Score (46); and shunt catheter type – either conventional, antibiotic or silver impregnated. Where not all variables are reported, regression will be conducted using the maximum number of covariables reported by all eligible studies.”  2. The sensitivity analyses have been updated as per reviewer one’s comments. These are now more specific. |
